# Supplementary material for: Scalable and energy-efficient task allocation in industry 4.0: Leveraging distributed auction and IBPSO
Source: PLoS One. 2025 Jan 16;20(1):e0314347. doi: 10.1371/journal.pone.0314347 (PMC11737662; doi:10.1371/journal.pone.0314347)
Supplement: Attachment — Submitted filename: Response to reviewers.docx [file pone.0314347.s001.docx]

Dear Reviewer 1，

I hope this message finds you well.

Thank you for your valuable and constructive feedback on our manuscript titled **"Scalable and Energy-Efficient Task Allocation in Industry 4.0: Leveraging Distributed Auction and IBPSO"**. We have carefully considered all of your suggestions and have made the necessary revisions to address each point.

Please find below our detailed responses to your comments:

1. **Low references in 2024**
   **Response:**
   We have added additional references from 2024 to ensure our manuscript reflects the most recent developments in the field. These references have been integrated throughout the text.
2. **Explanation of the structure of the paper at the end of the “Introduction” section**
   **Response:**
   We have revised the **Introduction** to include a clear explanation of the paper’s structure. The revised introduction outlines the background, research gap, objectives, and methodology, followed by a roadmap for the remaining sections.
3. **Keywords are not standard**
   **Response:**
   The keywords have been updated following the standard rules, avoiding repetition from the title and focusing on relevant terms. We have also ensured that no abbreviations are used.
4. **English presentation, typos, and long paragraphs**
   **Response:**
   We have reviewed and polished the manuscript for grammar, spelling, and overall presentation using Grammarly. Long paragraphs have been broken down for better readability and clarity.
5. **More detail about core contributions in the abstract**
   **Response:**
   The **Abstract** has been revised to provide more detail about the core contributions of the study, emphasizing the practical applications and innovations introduced in this research.
6. **Reduce the similarity index**
   **Response:**
   We have revised the text to reduce the similarity index, ensuring that the manuscript now complies with the authenticate requirements.
7. **Categorize research in a table in the Literature Review section**
   **Response:**
   We have added a table to the **Literature Review** section, categorizing previous studies and demonstrating the research gap that our paper addresses.
8. **Re-check all formulas**
   **Response:**
   All formulas have been re-checked for accuracy to ensure there are no errors in indices, typing, or concepts.
9. **Explanation of Figures and Tables**
   **Response:**
   We have reviewed all Figures and Tables and ensured that each one is clearly explained within the text and directly linked to the discussion.
10. **Add Figures in the Introduction**
    **Response:**
    We have added figures to the **Introduction** section to support the discussion and provide visual representation of key concepts.
11. **Managerial implications are missing**
    **Response:**
    We have included a new **Managerial Implications** section that highlights the practical applications of our research findings for industries implementing multi-robot systems under Industry 4.0.
12. **Check all references for correctness and duplication**
    **Response:**
    We have thoroughly reviewed all references to remove duplicates and ensure correct formatting according to the journal’s guidelines.

The revised manuscript with the necessary changes is attached, and all modifications have been highlighted in yellow for easy reference. We hope these revisions meet your expectations, and we look forward to your further feedback and technical comments.

Thank you once again for your time and effort in reviewing our manuscript. We greatly appreciate your insights and suggestions, which have helped improve the quality of our work.

Kind regards,

Dear Reviewer 2,

Thank you for your valuable feedback and insightful suggestions on our manuscript. We have carefully addressed each of your comments and made the necessary revisions. Below are our detailed responses to your feedback:

**1. Comparison with "A novel consensus protocol using facility location algorithms”**

**(2009 IEEE Control Applications, CCA & Intelligent Control, ISIC)**

**Response:** We have added a comparison between our approach and the consensus-based facility location algorithm. Our approach offers several advantages:

•Real-time Task Allocation: Unlike the consensus-based method, which optimizes global objectives, our approach focuses on dynamic and real-time task allocation. •Energy Efficiency and Scalability: We prioritize energy efficiency and scalability in industrial settings, which is a critical focus of our method.

•Decentralization: Both methods employ decentralized decision-making, but our IBPSO-enhanced algorithm brings greater adaptability and energy efficiency. We have discussed the pros and cons of both approaches, highlighting the advantages of our solution in handling dynamic and energy-efficient task allocations.

**2. Comparison with "Distributed energy resource management: All-time resource demand feasibility, delay-tolerance, nonlinearity, and beyond" (IEEE Control Systems Letters, 2023)**

**Response:** We have compared our work with distributed energy resource management techniques, emphasizing the following points:

•Energy Optimization: Our method focuses on real-time energy optimization within task allocation, while the referenced work addresses resource feasibility and delay tolerance.

•Localized Decision-Making: Both methods use localized decision-making, but ours is tailored for real-time task allocation in smart factories.

•Nonlinearity and Delays: While the referenced work focuses on nonlinearity and delays, our approach targets energy optimization and task performance. The comparison outlines the benefits of our IBPSO approach in highly dynamic industrial environments. **3. Computational Complexity of the Consensus-Based Algorithm**

**Response:** We have clarified the computational complexity of our decentralized task allocation algorithm. The complexity is of polynomial order, depending on the number of robots, tasks, and IBPSO iterations. This ensures scalability and efficiency, even in large-scale setups.

**4. Assumptions Regarding the Communication Network and Quantization**

**Response:** Our approach assumes a fully connected communication network with reliable communication between robots. While we do not explicitly model quantization in this study, we acknowledge its importance and suggest incorporating quantization effects in future work to better simulate real-world scenarios where communication bandwidth and noise could affect task allocation.

**5. Summary of the Proposed Algorithm in Algorithm Environment**

**Response:** We have summarized the main proposed algorithm in an Algorithm environment to improve clarity for readers. The key steps of the decentralized auction-based task allocation method with IBPSO are now clearly presented for easier understanding and implementation. Conclusion: We are grateful for your constructive feedback, which has helped us improve the clarity and depth of our manuscript. All changes have been made accordingly, and we look forward to your further comments. Thank you once again for your time and effort in reviewing our work.

Best regards,
